# End of lay postmortem findings in aviary housed laying hens

**DOI:** 10.1016/j.psj.2022.102332

**Published:** 2022-11-11

**Authors:** Páll Gretarsson, Käthe Kittelsen, Randi O. Moe, Guro Vasdal, Ingrid Toftaker

**Affiliations:** ⁎ Faculty of Veterinary Medicine, NMBU – Norwegian University of Life Sciences, Ås, Norway; † Animalia – The Norwegian Meat and Poultry Research Centre, Oslo, Norway

**Keywords:** pathology, end-of-lay, layer, poultry, cage-free

## Abstract

Good health and low mortality are constitutive elements of good animal welfare. In laying hens, mortality and pathological findings are usually reported as cumulative proportions from onset of lay to culling. However, knowledge of pathological lesions and causes of death specifically toward the end of the production period are scarce. This study aimed to investigate the occurrence of postmortem lesions and tentative causes of death in non-beak trimmed, end of lay hens, housed in multitiered aviary systems. A convenience sample of 48 flocks was recruited. In each flock, layers dead between wk 65 and 70 were necropsied in the field. In total, 482 layers were subjected to postmortem examination. The 4 most common pathological lesions were keel bone fracture (**KBF**) (92%), fatty liver (42%), emaciation (23%), and salpingitis (22%). Apart from keel bone fracture, the relative frequency of the pathological lesions variated between flocks, indicating that flock is an important factor. Common tentative causes of death were salpingitis (18%) and fatty liver hemorrhagic syndrome (**FLHS**) (13%). This study sheds light on health challenges aviary housed layers are facing end of lay, which is crucial knowledge in the development of preventive measures to secure good health and welfare.

## INTRODUCTION

Animal welfare has several definitions, depending on one's perspective, criteria and concept (Fraser, 2008). Fraser's (2008) 3 overlapping conceptions; affective states, natural living and basic health and functioning, represent one of the most acknowledged definitions of animal welfare today. Good health and low mortality are constitutive elements of good animal welfare with additional impact on farmers economy and sustainability (Fraser, 2008). The use of cage free systems, like aviary systems, is increasing in Europe and elsewhere (Schuck-Paim et al., 2021). These systems give the hens more opportunity for natural behavior, freedom of movement and choice (Lay et al., 2011; Stadig et al., 2015), however, they have also been reported to have higher mortality than cage systems (Aerni et al., 2005; Freire and Cowling, 2013; Weeks et al., 2016). Mortality is commonly used as a proxy for health and welfare monitoring in production animals. In laying hens, mortality is usually reported as cumulative mortality throughout the production period, from onset of lay to the time of slaughter or culling of the flock. Internationally, studies report that mortality in layers can range from 5% to 12% in cage-free systems, with highest mortality in free-range systems (Abrahamsson et al., 1998; Blokhuis et al., 2007; Fulton, 2017). A recent meta-analysis by Schuck-Paim et al. (2021) found that increased experience and knowledge on flock management for cage-free systems decreases mortality rates in aviary housing systems. In another metanalysis, Weeks et al. (2016) found an increase in predicted mean cumulative mortality throughout the production period of 3.9%, 7.4%, and 9.3% at the age 40, 60, and 72 wk respectively, for free range layers. In Norway, the mean cumulative mortality was 3.74% in cage-free layers (aviary indoor) at 71 wk of age in 2020 (Animalia, 2021). The vast majority (94%) of layers in Norway are kept in cage-free systems (Animalia, 2021). Of all flocks housed in cage-free systems, 84% are housed in aviary indoor systems (Animalia, 2021). Due to Norwegian legislations, the maximum housing limit is 7500 layers. Exceeding the limit requires permission from the authorities (Lovdata, 2004).

Common causes of mortality in laying hens are bacterial- or parasitic infections or causes related to egg laying (Fossum et al., 2009; Fulton, 2017), including salpingitis, salpingoperitonitis, egg yolk peritonitis, and egg impaction (Bisgaard and Dam, 1981; Jordan et al., 2005). In addition, behavioral problems like cannibalism may be an important mortality cause in all housing systems, however this tends to be more prevalent in cage-free systems (Cronin and Glatz, 2021). Severe feather pecking and cannibalism are considered to be abnormal behaviors, with multifactorial causes, that are still not fully known despite numerous studies on the matter (Cronin and Glatz, 2021). Main theories are for example, misdirection of foraging behavior (Huber-Eicher and Wechsler, 1997; Dixon et al., 2008), stress (Rodenburg et al., 2013) and dietary deficiencies (Kjaer and Bessei, 2013). Several countries practice beak trimming as a measure to reduce injuries from pecking behavior, however this has been prohibited in Norway since 1974 (Lovdata, 2001). Keel bone fracture (**KBF**) is one of the most common health problems in layers, with a prevalence of about 85 to 97% at end of lay (Rufener and Makagon, 2020). Fatty liver is another prevalent conditions in laying hens (Fulton, 2017). Fatty liver and KBF will usually not affect mortality, however, an excessive accumulation of fat in the liver can lead to hemorrhage, a fatal condition known as fatty liver hemorrhagic syndrome (**FLHS**).

There is an increasing interest within Europe to extend the production cycle for layers to 100 wk (Bain et al., 2016; Preisinger, 2018). Today, most flocks get replaced around 72 wk of age, mainly due to increased variation in egg quality at end of lay as well as a decrease in laying rate (Bain et al., 2016). These factors might be improved through selective breeding for a prolonged production cycle (Bain et al., 2016; Preisinger, 2018; Alfonso‐carrillo et al., 2021). However, with mortality increasing at end of lay (Weeks et al., 2016) it is important to assess the layers’ health and causes of death during the last weeks of the laying period before prolonging the production cycle, as a basis for planning preventive measures.

As mentioned, mortality and pathological findings are mostly reported as cumulative, and little is known about pathological lesions and causes of death in non-beak trimmed, aviary housed layers at specific time points of the production period, such as at end of lay. Understanding the health and welfare challenges that aviary housed, non-beak trimmed layers are facing end of lay is essential to secure the health and welfare of the birds, in particular if the production cycle is to be extended. Therefore, this study aimed to investigate the occurrence of different postmortem findings and tentative causes of death in non-beak trimmed, end of lay hens housed in aviary systems in Norway.

## MATERIAL AND METHODS

### Study Sample

This descriptive study is based on a convenience sample of 48 egg farms, each farm with one flock housed in indoor aviary system, recruited through the egg packing companies. Recruitment was based on the farmers willingness to participate. The included farms were located in three different geographical regions in Norway. Due to restrictions caused by both an Avian influenza (**AI**) outbreak and the COVID-19 pandemic, the sampling was not evenly distributed across regions. All layers within each flock were of the same hybrid. All layers were either Lohmann LSL or Dekalb white, the two most common hybrids in Norway (Animalia, 2020). The farmers were instructed to collect and freeze all layers found dead between 65 and 70 wk of age and mark them according to the week of death. Farm visits were conducted in production wk 70 to 76 by one of the 3 authors (P.G., K.K., G.V.) to gather production data and perform gross postmortem examination on all collected dead layers. Production data including hybrid, flock size at onset of lay, region and date of visit, was recorded at each visit prior to the postmortem examination. The visits were conducted from May 2020 to August 2021.

### Postmortem Examination

Dead layers were examined with in-field necropsy. The frozen birds were thawed on farm for one day prior to the postmortem examination. Age at death (in weeks) was recorded for each layer. Extensively cadaverous carcasses were not examined. Prior to the field work, a protocol for recording pathological findings during necropsy was made. The protocol included the following list of findings to be recorded: emaciation, mild fatty liver, moderate fatty liver, FLHS, peritonitis, hepatitis, salpingitis, impacted crop, egg impaction, vent pecking, toe pecking, and KBF. The presence or absence of each pathological finding, based on gross lesions, was recorded. Multiple conditions could be recorded for each layer, except conditions related to liver pathology where only one of the three categories (mild, moderate, FLHS) could be recorded. Mild fatty liver was recorded if parts of the layer's liver appeared with slightly yellow, diffuse discoloration. Moderate fatty liver was recorded if the entire liver was yellow, soft, enlarged and friable. FLHS was recorded if the layer presented a fatty liver and a blood clot in the abdominal cavity or on the ventral surface of the liver. Emaciation was recorded if the layer had a prominent keel and marked muscle atrophy, little to no abdominal fat and gelatinous epicardial fat. Prior to necropsy the keel was palpated, to compare palpation and necropsy results for KBF and calculate diagnostic accuracy. During necropsy, the number of KBF, along with the anatomical location were recorded (mid or caudal). The visceral surface of the keel bone was examined to record KBF. Transversal lines in the keel bone, with various degrees of callus, were recorded as KBF. The list of findings to be recorded also contained a nominal option for comments and tentative cause of death based on the necropsy findings.

### Data Management and Statistics

All observations were entered into a Excel spreadsheet (Microsoft Corporation, 2018). The data was later transferred to StataSE 16 (StataCorp, 2019) for cleaning and statistical analysis. A binary variable for KBF was created based on the variable for numbers of fractures on caudal tip of keel bone and number of fractures on mid keel bone. The variables “mild fatty liver,” “moderate fatty liver,” and “fatty liver hemorrhagic syndrome” were combined into one binary (0/1) variable called “fatty liver.” Descriptive statistics were performed both overall and on flock level. Diagnostic accuracy of palpation relative to necropsy to diagnose KBF was assessed for the three observers (P.G., K.K., G.V.).

### Ethical Statement

The study did not involve any handling on live animals, experimental manipulations, or invasive procedures. It was therefore exempt from approval of animal use by the Norwegian Food Safety Authority.

## RESULTS

### Study Sample

Overview of recorded production data is shown in Table 1. A total of 48 egg farms participated in the study. Two farmers had freezer malfunction and were therefore excluded from the study, resulting in 46 farms. The study sample consisted of 482 layers, with 2 to 25 necropsied layers per flock (mean 10, median 9). The flock size ranged from 5,300 to 19,004 layers with a mean of 7,911.5 and a median of 7,500. Of the 46 farms, 32 had a Lohmann LSL hybrid and 14 had a Dekalb White hybrid. The reported cumulative mortality, at date of visit, in the flocks ranged from 0.5 to 9.0% with a mean of 3.1%.

**Table 1:** Overview of collected production data from all flocks (n = 46).

Flock information	
Lohmann LSL (number of flocks)	32
Dekalb white (number of flocks)	14
Mortality; mean (range)	3.1% (0.5%–9.0%)
Flock size at onset of lay; mean (range)	7911.5 (5300–19004)

Overall and for three different observers (Ob 1–3).

### Postmortem Examination

Results from the postmortem examinations are shown in Tables 2 to 6. In total, 482 layers were necropsied. Of those, 356 (74%) were Lohmann LSL hybrids and 126 (26%) were Dekalb White hybrids. Several farmers continued to collect dead layers up until week 75, instead of stopping at wk 70. To increase the sample size, layers collected after wk 70 were included. This resulted in layers’ week of death ranging from 65 to 75 wk of age instead of 65 to 70 wk of age.

**Table 2:** Diagnostic accuracy of palpation as a diagnostic method for detecting keel bone fracture relative to necropsy.

	Overall (n = 482)		Ob 1 (n = 124)		Ob 2 (n = 88)		Ob 3 (n = 140)	
		95% CI		95% CI		95% CI		95% CI
Sensitivity	81.9%	78.0%–85.4%	60.2%	50.5%–69.3%	94.8%	87.2%–98.6%	96.3%	91.5%–98.8%
Specificity	82.1%	66.5%–92.5%	81.8%	48.2%–97.7%	81.8%	48.2%–97.7%	100.0%	54.1%–100%
Positive predictive value	98.1%	96.1%–99.2%	97.1%	90.1%–99.7%	97.3%	90.7%–99.7%	100.0%	97.2%–100%
Negative predictive value	28.6%	20.4%–37.9%	16.7%	7.9%–29.3%	69.2%	38.6%–90.9%	54.6%	23.4%–83.3%

**Figure 1:**
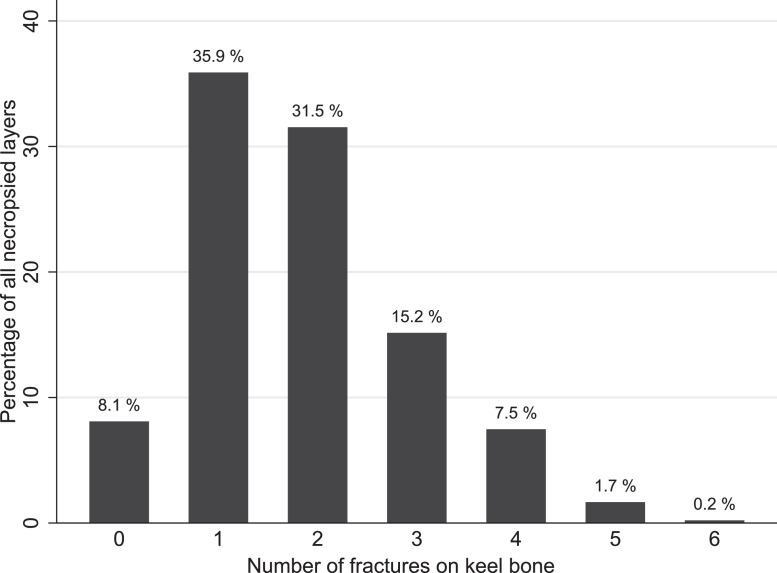
Relative frequency distribution of number of fractures of the keel bone per layer (n = 482) in a study investigating postmortem findings in aviary housed end of lay layers.

The most common pathological lesion was KBF; 443 (92%) of the layers had one or more KBF; 173 (36%) had 1 fracture and 270 (56%) had 2 or more fractures (Figure 1). The median number of KBF was 2, with a range from 0 to 6 per layer. At least 1 fracture was located on the caudal tip of the keel bone in all the 443 birds, and 23 (4.8%) layers had an additional fracture on mid keel bone. The frequency distribution of number of KBF per necropsied layer is shown in Figure 1. On flock level, the prevalence ranged from 60% to 100% with a median of 95%. The diagnostic accuracy of palpation as a diagnostic method for KBF detection, as well as the variation between the three observers (Ob 1–3), is shown in Table 2. Out of the 443 layers with KBF diagnosed from necropsy, 363 (Sensitivity: 82%) layers were correctly classified as such when palpated. Of the 39 layers with absence of KBF in necropsy, 32 layers (Specificity: 82%) were correctly classified with palpation. The observers did not palpate the same layers.

Fatty liver was the second most common lesion, found in 201 (42%) layers (flock median: 50%, flock range: 0%–100%). Of these, 101 layers were recorded with mild fatty liver, 34 layers with moderate fatty liver and 66 layers were recorded with FLHS. Emaciation was the third most common condition, found in 113 (23%) layers (flock median: 24%, flock range: 0%–75%). Only 6 layers were recorded with emaciation as the only pathological condition. The fourth most common pathological lesion was salpingitis, found in 104 (22%) layers (flock median 20%, flock range: 0%–80%). Peritonitis was recorded in 69 (14%) layers (flock median: 8%, flock range: 0%–72%). It was often comorbid with other lesions, with salpingitis as the most common one. Salpingitis and peritonitis were recorded together in 43 (9%) of all necropsied layers. Other lesions were observed in 10% or less of the cases, shown in Tables 3 and 4 (frequency of all layers) and Table 5 (frequency on flock level).

**Table 3:** Frequency table of gross pathological lesions of all necropsied layers (n = 482).

Lesion	Number of layers	Overall prevalence	95% CI		
Keel bone fracture	443	92%	89%	-	94%
Fatty liver, all combined	201	42%	37%	-	46%
Fatty liver by severity:					
*-mild*	*101*	21%*	17%	-	25%*
*-hemorrhagic syndrome*	*66*	14%*	11%	-	17%*
*-moderate*	*34*	7%*	5%	-	10%*
Emaciation	113	23%	20%	-	27%
Salpingitis	104	22%	18%	-	26%
Peritonitis	69	14%	11%	-	18%
Vent pecking	50	10%	8%	-	13%
Crop impaction	47	10%	7%	-	13%
Pecked toe	28	6%	4%	-	8%
Egg bound	16	3%	2%	-	5%
Hepatitis	8	2%	1%	-	3%

Italic: frequency within variable “Fatty liver, all combined.”

⁎ Percentage of all necropsied layers.

**Table 4:** Frequency table of gross pathological lesions of all necropsied layers divided into Lohmann LSL (n = 356) and Dekalb White (n = 126).

	Lohmann white					Dekalb white				
Lesion	Number of layers	Prevalence	95% CI			Number of layers	Prevalence	95% CI		
Keel bone fracture	324	91%	88%	-	94%	119	94%	89%	-	98%
Fatty liver, all combined	141	40%	34%	-	45%	60	48%	39%	-	57%
Fatty liver by severity:										
*-mild*	*74*	21%*	17%	-	25%*	*27*	21%*	15%	-	30%*
*-hemorrhagic syndrome*	*37*	10%*	7%	-	14%*	*29*	23%*	16%	-	31%*
*-moderate*	*30*	8%*	6%	-	12%*	*4*	3%*	1%	-	8%*
Emaciation	93	26%	22%	-	31%	20	16%	10%	-	23%
Salpingitis	78	22%	18%	-	27%	26	21%	14%	-	29%
Peritonitis	58	16%	13%	-	21 %	11	9%	4%	-	15%
Vent pecking	41	12%	8%	-	15%	9	7%	3%	-	13%
Crop impaction	44	12%	9%	-	16%	3	2%	1%	-	7%
Pecked toe	28	8%	5%	-	11%	0	0%	0%	-	3%^1^
Egg bound	11	3%	2%	-	5%	5	4%	1%	-	9%
Hepatitis	6	2 %	1%	-	4%	2	2%	0.2%	-	6%

1 One sided, 97.5% CI. Italic: frequency within variable “Fatty liver, alle combined.”

⁎ Percentage of all necropsied layers.

**Table 5:** Relative frequency of gross pathological lesions on flock level.

	Median	Range		IQR	
Lesion		Min	Max	25%	75%
Keel bone fracture	95%	60%	100%	85%	100%
Fatty liver, combined	50%	0%	100%	25%	57%
Emaciation	24%	0%	75%	8%	36%
Salpingitis	20%	0%	80%	11%	30%
Peritonitis	8%	0%	72%	0%	20%
Vent pecking	0%	0%	67%	0%	12%
Crop impaction	0%	0%	63%	0%	10%
Pecked toe	0%	0%	100%	0%	0%
Egg bound	0%	0%	50%	0%	6%
Hepatitis	0%	0%	11%	0%	0%

Proportions are based on necropsies of 482 layers found dead on farm from wk 65 to 75 in 46 farms.

**Figure 2:**
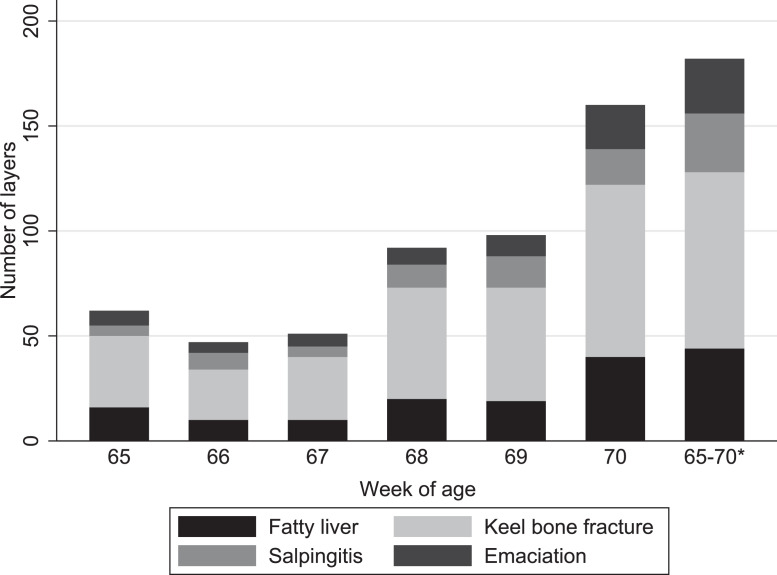
The 4 most common lesions in dead layers (n = 300) for each week of age from 65 to 70 wk, in a study investigating postmortem findings in aviary housed end of lay layers. Each layer can have more than one lesion. *Layers dead in the period 65 to 70 wk of age, lacking recording for specific week of age (n = 93 layers).

Distribution of the 4 most common lesions for each week of death is shown in Figure 2. Layers that died after wk 70 were excluded (n = 89) in the figure, as only some farmers collected layers beyond wk 70. Specific marking for week of death was lacking in 93 layers, resulting in their age at death to be recorded as 65 to 70 wk. The frequency of each lesion relative to the others remained similar for the most common lesions through the 5-wk period (Figure 2). Keel bone fracture was the most common lesion in every week, followed by fatty liver, salpingitis, and emaciation. There was an apparent increase in overall morbidity and mortality during the 5 wk period, however estimation of population values for these measures was not performed.

Tentative cause of death was recorded for each bird, based on which gross pathological lesion that most likely caused the mortality (Table 6). One layer had a missing record and was excluded from the overview of tentative cause of death, leaving 481 layers. The most common tentative cause of death was an unknown cause, recorded in 227 (47%) layers, followed by salpingitis, recorded in 86 (18%) layers, and FLHS, recorded in 66 (13%) layers. The rest of recorded tentative causes of death accounted for less than 10% (Table 6).

**Table 6:** Frequency of tentative cause of death for all necropsied layers (n = 481).

Tentative cause of death	Number of layers	Overall prevalence
Fatty liver hemorrhagic syndrome	66	13.7%
Unknown	227	47.2%
Salpingitis	86	17.9%
Emaciation	36	7.5%
Bleeding of unknown origin	14	2.9%
Fatty liver	11	2.3%
Egg bound	9	1.9%
Alimentary system	9	1.9%
Cannibalism	8	1.7%
Crop impaction	7	1.5%
Trauma	4	0.8%
Hepatitis	4	0.8 %

## DISCUSSION

This study describes the occurrence of different postmortem lesions and tentative causes of death in end of lay, non-beak trimmed layers housed in aviary systems in Norway. The four most common pathological lesions were, in decreasing order: KBF, fatty liver, emaciation and salpingitis.

The mean mortality in this study was 3.1%. This is in accordance to reported mortality in layers housed in cage-free systems in the Norwegian egg industry, which was 3.74% in 2020 (Animalia, 2021). However, this number is low compared to numbers reported from other studies on layers in similar housing systems (5–12%) (Abrahamsson et al., 1998; Blokhuis et al., 2007; Fulton, 2017). Another study reported mean mortality to be as high as 17.7% in nonbeak trimmed layers (Flock et al., 2005). The low mortality in our sample, compared to international studies, could be related to a number of factors such as differences in management, biosecurity practices, flock size, farm density, and disease situation. There are few outbreaks of infectious diseases in the Norwegian poultry industry (Animalia, 2021).

The most common lesion was KBF, recorded in 92% of the layers and it was the only lesion that was present in all the 46 flocks. The high prevalence of KBF at end of lay in this study is similar to other studies of aviary housed layers, assessed with necropsy (Rodenburg et al., 2008; Stratmann et al., 2015; Buijs et al., 2019; Thøfner et al., 2021). In an observation study from Danish flocks an overall prevalence of 90% in barn housed/aviary systems was reported in layers collected at depopulation (Thøfner et al., 2021). In an experimental study, Stratmann et al. (2015) reported a KBF prevalence ranging from 83% to 90.5% at 66 wk of age, across the 4 intervention groups (perches, platforms, ramps and control) all groups housed in an aviary housing system. Rodenburg et al. (2008) reported an overall prevalence of 97%, assessed with necropsy in aviary housed layers aged 59 to 63 wk of age. Buijs et al*.* (2019) reported a prevalence of fractures on the keel bone to be 85.1% in 75 wk old hens assessed with necropsy. The caudal tip was the most common location of KBF in the present study. This is in agreement with Thøfner et al. (2021) and Baur et al. (2020), who reported a fracture located on the caudal tip in more than 96% and 77% of all layers with fracture(s), respectively. The high prevalence of KBF and the potential pain it causes (Nasr et al., 2012), makes it an important welfare problem for commercial laying hens (Riber et al., 2018).

The method used to diagnose keel bone fracture varies between studies, and includes palpation, necropsy, computed tomography, radiography, or a combination of several methods (Rufener and Makagon, 2020). In the current study, each keel bone was palpated prior to necropsy. Comparing the palpation results and necropsy findings showed that both the sensitivity and specificity of palpation was 82%. This is in line with several other studies that have reported a low accuracy of palpation as a diagnostic method for KBF (Richards et al., 2011; Casey-Trott et al., 2015; Buijs et al., 2019; Rufener and Makagon, 2020; Thøfner et al., 2021). The imperfect accuracy should be considered when interpreting results from studies solely relying on palpation. As our results on KBF are based on necropsy, we consider the internal validity as high for the reported KBF occurrence.

A total of 42% layers were recorded with fatty liver, ranging from mild via moderate to the most severe which is FLHS. In about half of the layers recorded with fatty liver, the condition was mild. It is not uncommon for layers to have a mild form of fatty liver, due to high demand on lipogenesis, which in birds mainly occurs in the liver (Zaefarian et al., 2019). Of all the layers recorded with fatty liver in our study, 33% had FLHS. Birds with FLHS will die due to a rupture of the liver capsule which is followed by an acute blood loss. The exact etiology of FLHS is unknown but is believed to be related to diet and estrogen levels (Zaefarian et al., 2019; Shini et al., 2020). It has been shown to be a more common cause of death in cage systems than cage-free systems (Shini et al., 2019). Wang et al. (2019) reported a FLHS prevalence of 0% in cage-free layers in a random sample of culled flocks at end of lay. The sample in the current study consisted of layers dead on farm through a 10-wk period and it was thus expected to find a higher prevalence. The occurrence of FLHS can increase with age (Dong and Tong, 2019). However, reports on the relationship between age and FLHS are scarce, nevertheless our results suggest that FLHS is a common cause of mortality in aviary housed layers aged 65 to 75 wk.

Salpingitis was recorded in 22% of the layers. When salpingitis and peritonitis appeared together, they were recorded as two single lesions, and not as salpingoperitonitis. Salpingitis can be caused by ascension of bacteria from cloaca or secondary to egg binding (Jordan et al., 2005; Landman et al., 2013). Salpingitis has previously been reported as a common pathological lesion in commercial layers, in line with the current study (Fulton, 2017; Wang et al., 2019). A study of postmortem findings in culled flocks at end of lay reported lesions in the reproductive tract to be the second most prevalent lesion, with a higher prevalence in barn housed and organic/free range layers (3.12% and 4.56%, respectively) compared to enriched cages (2.84%) (Wang et al., 2019). Those numbers are much lower than the current study (22%), however, as discussed earlier with the FLHS number, Wang et al. (2019) examined a random sample of layers from culled flocks at end of lay whilst the current study examined layers dead on farm during a 10 wk period end of lay. Fulton (2017) reported salpingitis to be one of the five most common lesions in a study examining layers dead on farm throughout the production cycle. The prevalence of salpingitis may be underestimated in the present study, as the diagnosis was based on gross pathological lesions and no bacteriology was performed. The true prevalence of salpingitis may have been higher than the 22%, since 46 layers had been subjected to cannibalism and lesions in the oviduct could not be assessed.

Emaciation was one of the four most common conditions found in our sample. Emaciation is often secondary to a primary cause (Gregory and Robins, 1998; Reimers et al., 2019). Of the 113 layers recorded with emaciation in our study, only 6 were recorded solely with emaciation. Apart from KBF (present in almost all birds), other common lesions comorbid with emaciation were fatty liver, vent pecking, and salpingitis. Reimers et al. (2019) reported intussusception of the proventriculus as a cause of emaciation and sporadic mortality. Their study sample was, however, from older layers (aged 108–212 wk), and the first case occurred in a 110-wk-old flock, implying that it is unlikely to find this condition in a commercial flock. In the current study, pathological lesions in the alimentary tract were not recorded. One study reported emaciation to be highest in barn and free-range systems, although emphasizing that the prevalence was high in all examined housing systems (conventional cage, furnished cage, barn, and free-range) (Sherwin et al., 2010).

For the four most common lesions, the frequency of each lesion relative to the others remained the same throughout the study period (65–70 wk), with an overall trend of increasing morbidity and mortality with age. However, as not every farmer collected all dead layers, and not all layers were marked with specific age at death the study sample was not suitable for accurate inference on total morbidity and mortality. Further research is needed to assess the association between age, morbidity and mortality in layers.

The relative frequency of the pathological lesions varied between flocks. The lesions with the largest variations between flocks were fatty liver, emaciation, peritonitis and salpingitis. This indicates that flock is an important factor for these lesions and that it might be possible to apply measurements for improvement on farm, such as improving management, feeding and production routines. On the other hand, KBF was common in every flock, indicating that a strong association between the risk of KBF and herd health management is unlikely, and that the etiologic factors are found elsewhere.

All postmortem examinations in the current study were performed in-field and records of lesions are based solely on gross pathology, bacteriology, and histology were not included. This approach is practical and cost-effective, however, it can result in underestimation of lesion prevalence and also poses a limitation to the accuracy of the recorded tentative cause of death. In our sample 47% layers were recorded with an unknown cause of death. Of those, 17% layers were missing either intestines, oviduct, or both. Cause of death in these cases are likely due to cannibalism, however, cadaverous changes made it difficult to distinguish primary cannibalism from secondary cannibalism. They were thus recorded with unknown cause of death and a comment on missing intestines and/or oviduct. If missing intestine and/or oviduct had been included in cannibalism as tentative cause of death, this would result in 11% layers potentially dying of cannibalism making it the fourth most common cause of death in the sample. Fossum et al. (2009) reported that cannibalism was responsible for 18.6% of layer mortality in litter-based systems (single-tiered and aviary), in non-beak trimmed layers from 18 to 78 wk of age. Cannibalism is previously reported as common in cage-free systems (Fossum et al., 2009; Cronin and Glatz, 2021) and in flocks with intact beaks (Abrahamsson and Tauson, 1995), in line with the current study. Other common tentative causes of death in the present study were salpingitis (18%) and FLHS (14%). Salpingitis and FLHS were previously reported as common causes of death in studies by Fulton, 2017, Fulton, 2019, salpingitis being the fifth most common cause of death with a prevalence of 4.7% (Fulton, 2017). However, the study included several causes that were not included in the current study; egg yolk peritonitis, hypocalcemia and gout, which all preceded salpingitis as a cause of death (Fulton, 2017).

The number of dead layers collected by farmers varied from 2 to 25, per farm, with a median of 9 layers. We cannot be certain that the farmers collected all layers that died as they may have forgotten or ran into practical problems with freezer capacity. This needs to be taken into consideration when interpreting the data on flock-level. Other limitations are that the selection of farms was based on a convenience sample. This may hamper the generalization of study results. However, the selected farms do not differ from the general population of Norwegian farms in important characteristics such as hybrid, production system and flock size. Thus, we believe the reported pathological findings to have acceptable external validity for Norwegian aviary housed layers.

The results from this study shed light onto health conditions Norwegian aviary housed layers are facing at end of lay. This knowledge can be used as a basis for further research investigating risk factors for each pathological lesions and the association with the layers’ environment and management, with the ultimate goal to improve health and welfare. More importantly it can be used to target preventive efforts against the most common diseases.

## CONCLUSION

In this study of aviary housed, non-beak trimmed commercial layers, the 4 most common pathological lesions were KBF (92%), fatty liver (41%), emaciation (23%), and salpingitis (22%). Common tentative causes of death were salpingitis (18%) and FLHS (14%). Apart from KBF, the relative frequency of the pathological lesions variated between flocks, indicating that flock is an important factor. The study also found a low accuracy of palpation as a diagnostic method for keel bone fractures, emphasizing the need to be cautious when making inference on prevalence and severity of keel bone fractures using palpation. The results are an important contribution to essential knowledge to secure the health and welfare of layers at end of lay, especially upon a possible extension of the production cycle.
